# Ecological restoration alters microbial communities in mine tailings profiles

**DOI:** 10.1038/srep25193

**Published:** 2016-04-29

**Authors:** Yang Li, Zhongjun Jia, Qingye Sun, Jing Zhan, Yang Yang, Dan Wang

**Affiliations:** 1 School of Resources and Environmental Engineering, Anhui University, Hefei, Anhui Province, China; 2 State Key Laboratory of Soil and Sustainable Agriculture, Institute of Soil Science, Chinese Academy of Sciences, Nanjing, Jiangsu Province, China

## Abstract

Ecological restoration of mine tailings have impact on soil physiochemical properties and microbial communities. The surface soil has been a primary concern in the past decades, however it remains poorly understood about the adaptive response of microbial communities along the profile during ecological restoration of the tailings. In this study, microbial communities along a 60-cm profile were investigated in a mine tailing pond during ecological restoration of the bare waste tailings (BW) with two vegetated soils of *Imperata cylindrica* (IC) and *Chrysopogon zizanioides* (CZ) plants. Revegetation of both IC and CZ could retard soil degradation of mine tailing by stimulation of soil pH at 0–30 cm soils and altered the bacterial communities at 0–20 cm depths of the mine tailings. Significant differences existed in the relative abundance of the phyla *Alphaproteobacteria*, *Deltaproteobacteria*, *Acidobacteria*, *Firmicutes* and *Nitrospira*. Slight difference of bacterial communities were found at 30–60 cm depths of mine tailings. Abundance and activity analysis of *nifH* genes also explained the elevated soil nitrogen contents at the surface 0–20 cm of the vegetated soils. These results suggest that microbial succession occurred primarily at surface tailings and vegetation of pioneering plants might have promoted ecological restoration of mine tailings.

Revegetation has been widely exploited to control environmental hazards associated with mine tailings containing polymetallic sulfides because plant community development can effectively increase the content of organic matter^1^,^2^,^3^,^4^,^5^ and nutrients in the tailings^4^,^6^,^7^,^8^; furthermore, plant community development is likely to inhibit the oxidation of polymetallic sulfides in tailings^9^,^10^. Microbial communities have long been believed to be the key drivers for polymetallic sulfide transformation, but the role of microbial communities during the ecological restoration of mine tailings remains largely unknown.

Ecological restoration of mine tailings by revegetation provides a model system for investigating biotic interactions below and above ground^9^,^11^. Microorganisms have been shown to rapidly respond to environmental change during the restoration process in an ecosystem of tailings. Microorganisms may be possible bio-indicators for monitoring soil ecosystem functions in close association with changes in the physicochemical and biological conditions during the ecological restoration of mine tailings^10^,^12^. For example, nitrogen fixation is often considered as an indicator for ecosystem quality because the deficiency of nitrogen in mine tailings usually necessitates rapid growth of microorganisms capable of nitrogen fixation^13^,^14^,^15^. Moreover, heterotrophic microorganisms are involved in the establishment of ecological vegetation, and the complex plant-microorganism interactions play an important role in sustaining physical structures in soil and in nutrient cycling^16^,^17^. To date, studies on microorganisms in tailings have primarily focused on the microbial biomass, function and activity^3^,^12^,^18^,^19^,^20^. However, few researchers have paid close attention to the change in microbial communities along tailings profiles. Although many investigations have been conducted on changes in the structure, composition and diversity of microbial communities during revegetation using methods based on molecular biology^1^,^2^,^21^,^22^, phospholipid fatty acids (PLFAs)^3^,^11^,^23^, community-level physiological profiles (CLPPs)^18^, etc., the current knowledge on the relationship between plants and soil microorganisms is still superficial due to limitations in these research methods. Pyrosequencing of the total 16S rDNA genes presents a powerful technique for analyzing the composition of microbial communities in complex environments with unprecedented coverage^9^,^24^,^25^,^26^,^27^,^28^.

China is most likely the country with the largest number of heavy metal mine tailings ponds, and mining activities have produced 20,000 km^2^ of mine tailings wastelands. In these mine tailings wastelands, a large amount of mine tailings containing polymetallic sulfides are stacked in several mine areas, such as Tongling^7^,^8^, which is an important mining city in China. To reduce the environmental risks associated with stacked copper mine tailings, ecological restoration by plant revegetation is usually carried out. Natural *Imperata cylindrica* and artificial *Chrysopogon zizanioides* were established on the Shuimuchong wasteland of copper mine tailings in Tongling (built in 1990). Both of the plant species are growing well as tolerant pioneer plants, and these plants are widely used for ecological restoration in tailings ponds^29^,^30^. Revegetation has been reported as main reason altered the soil chemical composition and bacterial community diversity in mine tailings^2^,^6^,^9^,^14^ and mine affected areas^3^,^11^,^17^. Most reports paid more attention to that the structure and abundance of bacterial community, especially functional bacteria, were changed by amendment strategies^2^,^9^ and primary succession^6^,^14^. Based on this, we expect to compare the soil chemical composition and bacterial community diversity in the soil profiles, which was used to further investigate the depths that largest influenced by revegetation. It was of great importance for restoration and administration of mine tailings. And it was also of value for the analysis of nitrogen-fixing ability of the bacteria which was used to meet the nutritive requirements for plant growth in a nutritionally deficient habitat. The objective of this study was to investigate the response of the bacterial community structure to plant species on profiles and to identify the factors controlling the structure of the bacterial community.

## Results

### Physicochemical properties

**Figure 1:**
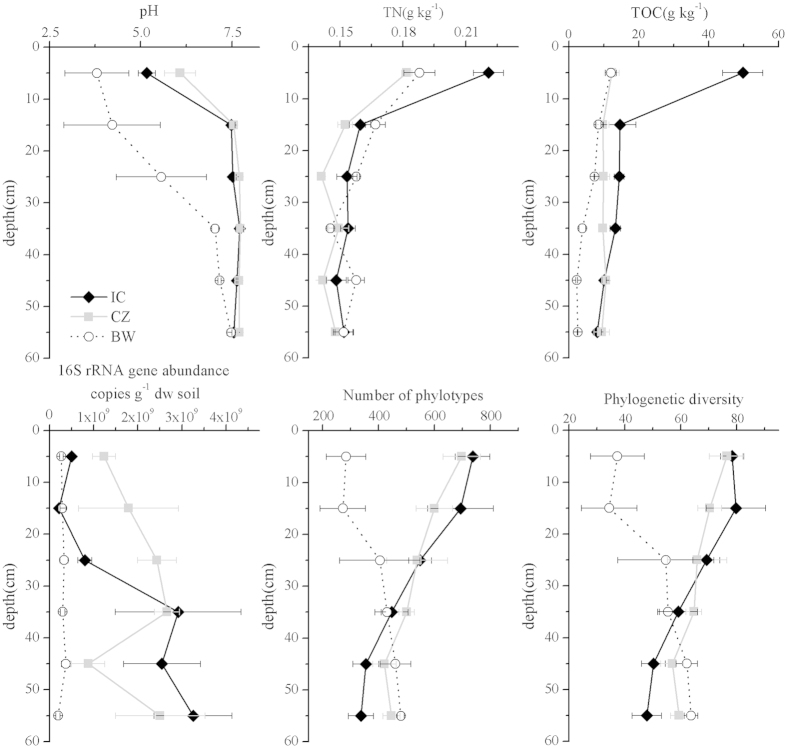
Soil chemical and biological properties in the copper mine tailings. The error bars show the standard error of the three subsamples for each sample of the tailings. TOC, total organic carbon; TN, total nitrogen.

Revegetation increased the pH value in the surface horizons at 0–30 cm when compared to bare wasteland, and no significant difference was observed in the fields between IC and CZ (Fig. 1). TOC and TN were significantly enhanced only in the field with IC at 0–10 cm. For heavy metals (As, Cu, Fe, Pb, Zn), revegetation only decreased the As content in the horizons at 0–60 cm and increased the Pb content in the horizons at 20–60 cm and the Zn content in the surface horizons at 0–20 cm (Supplementary Fig. S1). In addition, the CZ revegetation increased the 16S rRNA gene abundance throughout the profiles while the stimulation of 16S rRNA genes was observed only at the deep horizons at 30–60 cm of IC revegetation soil.

The mean number of high-quality sequences per sample was 3 648, ranging from 1 239 to 7 649. We identified a total of 6 909 distinct OTUs (at 97% similarity) in the dataset. Rarefaction curves of all samples at species level approached saturation (Supplementary Fig. S2), indicating that the sequencing depth is enough to the subsequent analysis. The number of phylotypes per sample ranged from 123 to 842. The revegetation increased the phylotypes and phylogenetic diversity in the surface horizons at 0–20 cm, and significant differences in phylotypes and phylogenetic diversity were not observed between plant species (Fig. 1).

### Changes in microbial community structure

**Figure 2:**
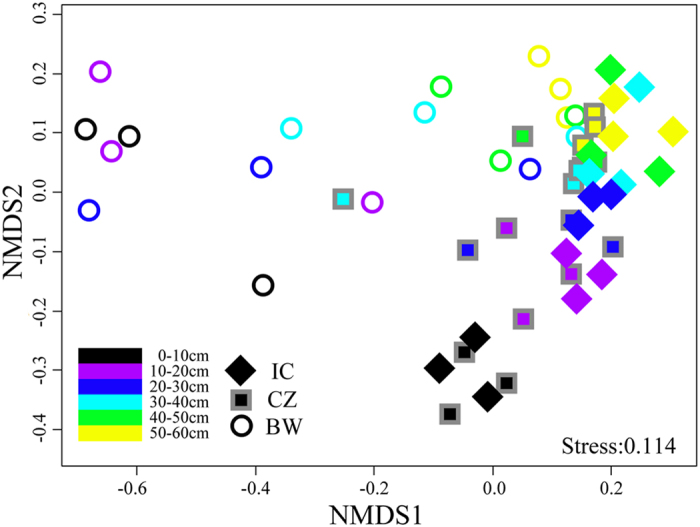
Ordering of the bacterial community composition by non-metric multidimensional scaling (NMDS) using the Bray-Curtis distance index. The various colors indicate different depths; n = 3. The variance explained by the two first axes is shown in the graph.

**Figure 3:**
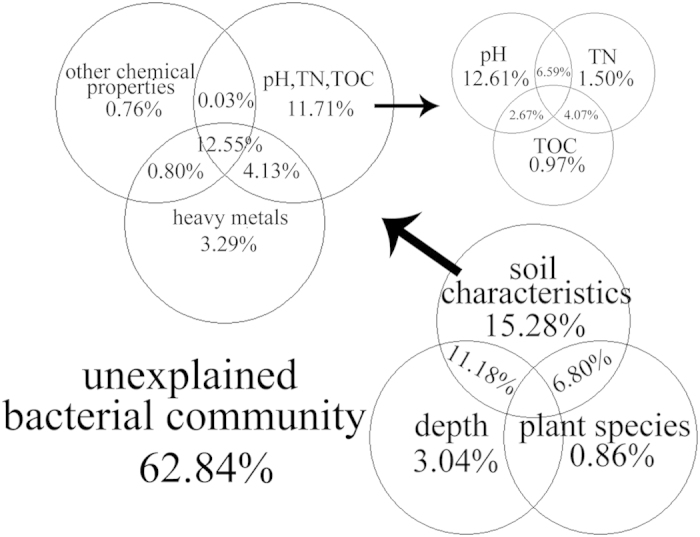
Variation partitioning analysis (VPA) was used to determine the effects of soil characteristics, plant species, and depth and interactions between these parameters on the structure of the bacterial community. Circles without overlap showed the percentage of variation explained by each factor alone. The overlap region of two or three circles displayed the explanation of variation between two or three of these factors.

The ordering of samples by non-metric multidimensional scaling (NMDS) based on their phylotype composition and using the Bray-Curtis similarity measure (Fig. 2) showed a separation of samples by depth in the tailings. Significant differences in soil microbial communities were observed in the fields between the revegetation area and the bare wasteland (BW) for the top 0–20 cm of the profiles, while no statistically significant difference was observed between IC and CZ vegetation (Fig. 2 and Table S1). VPA showed that soil parameters(especially pH) rather than plant species and depth were the main factor influencing the bacterial community (Fig. 3). Redundancy analysis (RDA) also showed that soil parameters were the main dimension to the bacterial community structure (Supplementary Fig. S3). The relationship between soil properties and phylogenetic diversity was further inferred by using the Bray-Curtis dissimilarity index (Supplementary Fig. S4), showing a close correlation between soil pH, TN, TOC and the shift in microbial community structure.

**Figure 4:**
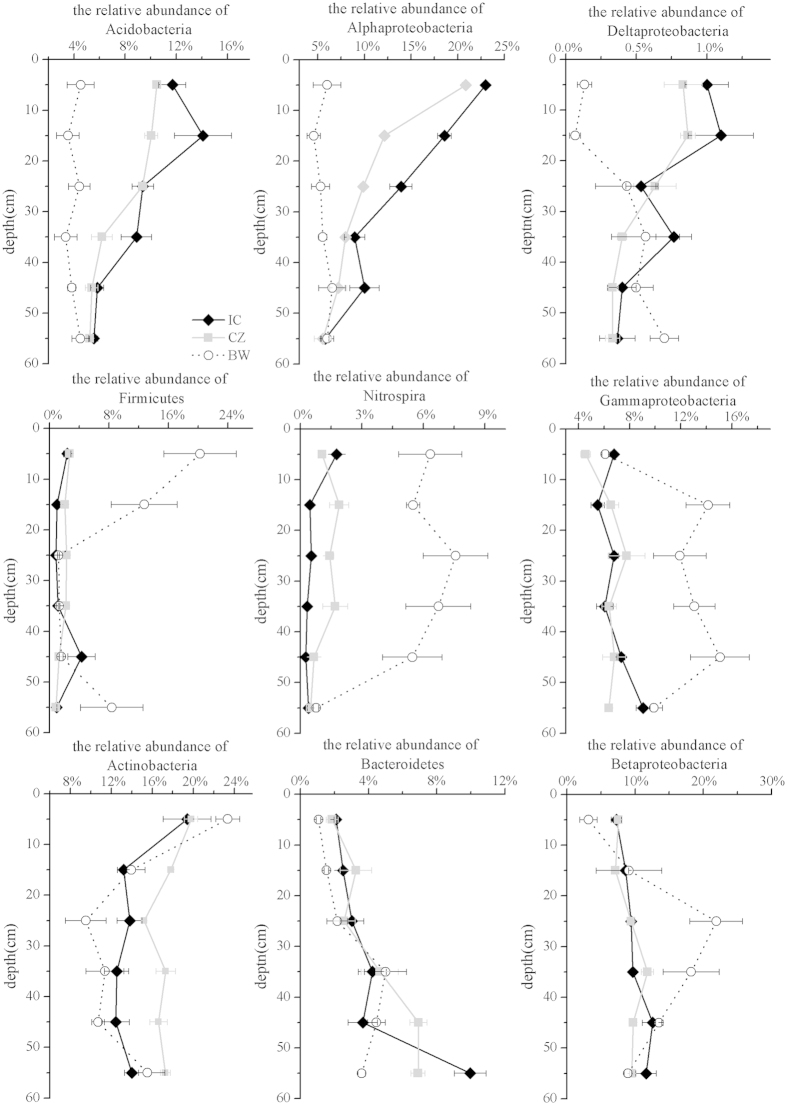
Relative abundance (percentage) of the main identified bacterial taxonomic groups, i.e., phyla *Acidobacteria*, *Actinobacteria*, *Bacteroidetes* and *Firmicutes*, classes *Alphaproteobacteria*, *Betaproteobacteria*, *Gammaproteobacteria* and *Deltaproteobacteria* (within the *Proteobacteria* phylum). For each tailings sample, the relative abundance of the sequences assigned to a given taxonomic unit was calculated for each of the three subsamples, and the average value was then used to represent the relative abundance of each tailings sample. The error bars show the standard error of relative abundance of the three subsamples for each tailings sample.

The high-quality sequence reads were taxonomically assigned to 22 phyla using the RDP-Classifier. The 6 numerically dominant phyla (with relative abundances higher than 1%) accounted for 68.87% of the total sequence reads (unclassified bacterium accounted for 26.66% of the total sequence), including *Proteobacteria*, *Actinobacteria*, *Acidobacteria*, *Firmicutes*, *Bacteroidetes* and *Nitrospira* (Fig. 4). When compared to the bare wasteland, the relative abundance of *Acidobacteria*, *Alphaproteobacteria* and *Deltaproteobacteria* was significantly higher in the field vegetated with IC and CZ at the top 0–20 cm of the profiles, while no other significant differences were observed for the above phyla at depths of 20–60 cm in the profiles. A contrasting pattern was observed for *Firmicutes* (Fig. 4). *Nitrospira* was observed in the profile horizons of 0–50 cm and *Gammaproteobacteria* in the profile horizons of 10–50 cm; moreover, no significant difference was found in any of the dominant phyla or classes within *Proteobacteria* between two such plant species.

### Distribution of nitrogen-fixing bacteria

**Figure 5:**
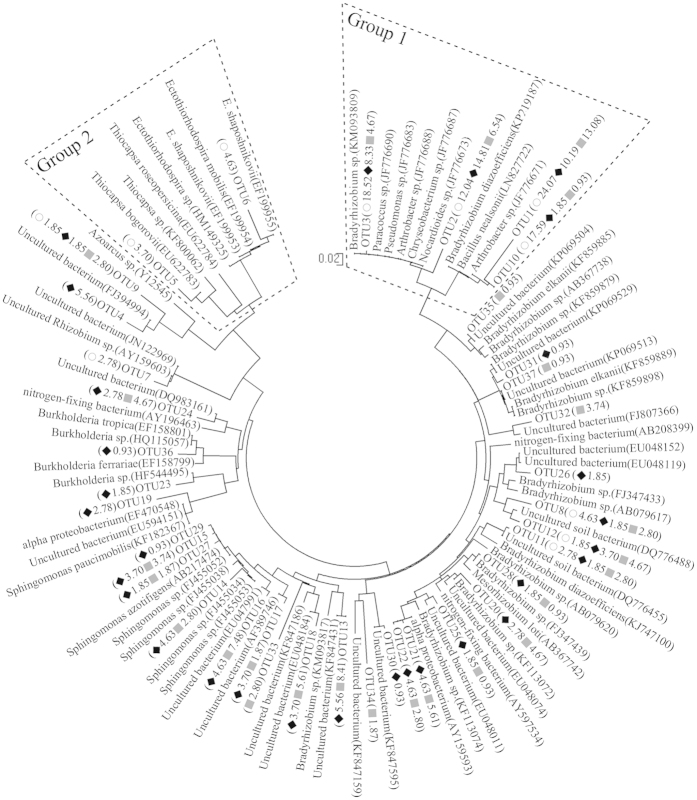
Phylogenetic tree of nucleotide *nifH* sequences in the top 20 cm of tailings. Additional symbols show the relative frequency (%) of a sequence in the respective clone libraries (○, BW; ◆, IC; ◼, CZ).

Ecological vegetation apparently stimulated the abundance of functional genes when compared to the bare soil, while the *nifH* genes showed higher abundance than red-like and green-like *cbbL* genes in the vegetated fields (Supplementary Fig. S5c). Meanwhile, phylogenetic analysis of the *nifH* genes indicated apparent nucleotide divergence of functional makers for biological nitrogen fixation between re-vegetated soil (IC and CZ) and bare wasteland (BW) in the surface tailings (Fig. 5). However, most of the *nifH* sequences displayed very low levels of similarity to *nifH* sequences of known diazotrophs. Group 1 was the unclassified group, and the *nifH* sequences of this group were the most homologous to the sequences of different bacteria (such as *Arthrobacter* belonging to *Actinobacteria*, *Bacillus* belonging to *Firmicutes* and *Bradyrhizobium* belonging to *Proteobacteria*). This group accounted for the majority of the *nifH* sequences of BW, IC and CZ. Group 2 was likely to belong to *Gammaproteobacteria* and was only detected in BW. Meanwhile, the sequences expected for Group1 and Group 2 were primarily distributed in the revegetation area and may belong to *Alphaproteobacteria*. All of these observations showed that direct revegetation changed the abundance (Supplementary Fig. S5c) and structure of the nitrogen-fixing bacteria in the tailings, and the additional species were related to *Alphaproteobacteria*. It has long been recognized that *Arthrobacter* within *Actinobacteria* are capable of biological nitrogen fixation^31^ (Fig. 5), which is also mainly distributed in IC and CZ based on the pyrosequencing results of 16S rDNA genes (Supplementary Fig. S6a). However, more members in phylogenetic analysis of the *nifH* gene did not observed in pyrosequencing results, which might due to the difference of genes fragments and experiment measures. However, no significant difference was found in the nucleotide *nifH* sequences between different plant species (Fig. 5, Supplementary Fig. S5c) or in the transcriptional *nifH* sequences in the rhizosphere (Supplementary Fig. S7). Compared to DNA based libraries, some *nifH* gene such as OTU4, OTU8, OTU9, OTU10, OTU11, OTU17, OTU18, OTU19, OTU22, OTU23, OTU28, OTU29, OTU30, OTU31, OTU36 and OTU37 with lower relative abundance of *nifH* gene did not exhibit in phylogenetic tree of transcriptional *nifH* sequences in rhizosphere. The *nifH* sequences in Group 1 of phylogenetic tree of nucleotide *nifH* sequences also showed high expression and also displayed as the main transcriptional *nifH* sequences in the rhizosphere.

## Discussion

To the best of our present knowledge, this report is the first to elucidate the shift in microbial communities under different plant species in tailings profiles. Our results show that revegetation improved the surface structure and diversity of the microbial community in the environment of mine tailings. Similar results have been reported in other studies of ecological restoration in copper mine tailings^2^,^6^,^9^,^14^, lignite mine areas^11^, post-mining afforested soils^3^ and a San Pedro River mine tailings site^17^. Moreover, pioneer plants also influence the structure and function of the microbial community in other extreme environments such as glaciers^15^ and semiarid ecosystems^19^. The selection of plant species has significant consequences for microbial communities and ecosystem functions in the topsoil, according to previous studies^1^,^3^,^27^. However, no significant difference in the microbial community diversity or structure was found between the two pioneer plants considered in this work. Most interestingly, a differential effect of revegetation on the diversity and structure of microbial communities was observed at different horizons of the profiles, possibly resulting from weakly varying soil physicochemical properties that were affected by tailings revegetation with different plant species^32^. The most pronounced changes in microbial communities may occur within the top 20 cm of the tailings, where many surface-dwelling microorganisms thrive^32^,^33^.

Our research showed that the revegetation may have resulted in a higher diversity of bacterial communities in the top20 cm (Figs 1 and 2 and Table S1), suggesting that the establishment of plants favors the growth of diverse microorganisms, as previously reported^2^,^9^. In parallel, our research demonstrated that revegetation may either directly or indirectly drive shifts in the bacterial communities around tailings, increasing the relative abundance of *Acidobacteria*, *Alphaproteobacteria* and *Deltaproteobacteria* and decreasing the relative abundance of *Firmicutes* in the surface horizons at 0–20 cm, *Nitrospira* in the horizons at 0–50 cm and *Gammaproteobacteria* in the horizons at 10–50 cm (Fig. 4). Previous studies have also shown the effects of plant colonization on these microbial phylotypes. For example, plants have led to an increase in soil C and N content, thereby favoring *Proteobacterial* taxa^21^,^25^,^34^, consistent with our report on the revegetated tailing site. It is thus likely that these microbial phylotypes play a vital role in the C and N cycle^19^,^34^. In addition, although *Acidobacteria* has been reported as dominant in several acidic environments^24^ and unvegetated soils^34^, the *Acidobacteria* taxa is genetically and metabolically diverse, having the capability to degrade cellulose and other compounds and is commonly dominant in forest soils^35^,^36^,^37^. Moreover, a higher abundance of *Firmicutes* has been found under extreme conditions^19^,^38^, in part due to their tolerance to very low substrate availability^19^,^38^. In parallel, only two genera (*Leptospirillum* and *Nitrospira*) belonging to the phylum *Nitrospira* were found in this study, and the effect of revegetation on the relative abundance of the phylum *Nitrospira* was primarily reflected by the decreased relative abundance of *Leptospirillum* (Supplementary Fig. S8). This genus may be associated with iron and sulfur oxidation, and the decreased relative abundance of this genus also indicates that revegetation inhibits the process of biological oxidation of the tailings.

In parallel, the microbial community may be attributed to vegetation development that facilitated soil physicochemical changes in barren mine tailings characteristics. For instance, acidic environments have been shown to be alleviated in association with organic matter accumulation^1^,^2^,^3^,^4^,^5^. Nitrogen in the tailings may also be attributed to the decomposition of plant litter and residues in the tailings fields, in addition to diazotrophs^4^,^6^,^7^. More strikingly, the increase of pH, TOC and TN in the surface tailings provided a basis for the increase of diversity in the bacterial community^25^ and structure^39^. The importance of environmental factors in shaping microbial communities has been established by a number of studies^3^,^18^,^26^,^36^,^40^. In this study, we found that the phylogenetic distance between bacterial communities was significantly correlated with soil characteristics, similar to previous studies revealing that soil characteristics, rather than plant species, are major drivers of their bacterial communities^28^,^40^. Chodak and Niklińska^18^ also found that tree species did not affect soil properties and had only a weak effect on community-level physiological profiles. In particular, pH might exert a direct stress on bacteria with certain pH levels, selecting some bacterial taxa over others^41^, and such major factors have also been shown to influence microbial communities in other mine wastelands^26^,^42^ and mining districts^3^,^12^. Apparently, revegetation can effectively inhibit the decline of pH and the oxidation of polymetallic sulfides in tailings. Although Li *et al.*^9^ considered that plants may accelerate the weathering of tailing minerals in revegetated tailings, revegetation plants increased the relative abundance of *Alphaproteobacteria* and decreased the relative abundance of *Firmicutes* and *Nitrospira*, all of which might significantly change as the oxidation progresses^24^,^26^ with the decreased pH. In parallel, the organic matter and nutrients coming from both litter and exudates have also been widely reported to influence individual taxa and/or the overall microbial community structure^18^,^23^,^40^,^42^,^43^,^44^ that supplies energy and nutrition for heterotrophic microorganisms. In addition, the RDA results (Supplementary Fig. S3) indicated that soil parameters had a significant effect on the microbial community structure. Meanwhile, VPA only demonstrated that heavy metals had a weak but significant effect on the microbial community structure, which might be related to the bacterial tolerance to heavy metals^20^,^25^. Overall, these results highlight the important role of revegetation in improving soil properties (especially pH), shaping the bacterial community structures and promoting further development of the microbes.

Low nitrogen content set a significant limit to the growth of plants in the tailings^13^. In addition to nitrogen in wastelands of copper mine tailings coming from plant litter and root exudates^15^, the nitrogen assimilation was primarily driven by soil diazotrophs including rhizobium and free-living nitrogen-fixing bacteria. Bare wasteland seems to have also been colonized by various nitrogen-fixing bacteria to relieve the nitrogen constraint in the tailings ecosystem and to provide nitrogen for other microorganism growth. It is possible that most of the nitrogen-fixing bacteria were autotrophic and related to iron and sulfur oxidation (such as OTU5 and OTU6 related to *Thiocapsa* and *Ectothiorhodospira,* respectively). Both *Thiocapsa* and *Ectothiorhodospira* have been reported as nitrogen-fixers^45^,^46^. Revegetation indeed increased the number (Supplementary Fig. S5) and diversity (Table S3) of diazotrophs in the tailings profiles, and certain free-living nitrogen-fixing bacteria related to *Alphaproteobacteria* increased (Fig. 5). Vegetating non-legumes have been reported to be capable of improving free-living nitrogen-fixing bacteria in tailings. Knelman *et al.*^15^ also showed a higher abundance of N_2_-fixing microorganisms in vegetated soils, and the structure of the nitrogen-fixing bacteria improved with the succession of vegetation in the tailings^14^. However, the abundance and structure of nitrogen-fixing bacteria among different pioneer plants showed no significant difference, similar to the results for the expression of functional genes (Supplementary Fig. S7; transcript abundance: IC: (1.55 ± 0.51) × 10^6^ copiesg^−1^ dry soil; CZ: (1.06 ± 0.09) × 10^6^ copiesg^−1^ dry soil; t-test: *P* = 0.380). In summary, we speculate that revegetation may increase the nitrogen content through enriched communities of nitrogen-fixing bacteria and observed a distinct increased nitrogen content in the surface tailings for IC, revealing that IC revegetation might be more suitable for accumulating nutrients in the tailings.

The iron- and/or sulfur-oxidizing bacteria living in the tailings played an important role in the oxidation of metallic sulfides, especially for pH <3^24^,^26^,^47^,^48^. Previous studies have indicated a high abundance of sulfur-oxidizing bacteria in the extensive range of pH > 3^49^, which resulted in the further oxidation of metallic sulfides in the tailings^22^. Preventing the oxidation of metallic sulfides in the tailings is very important for plant survival^22^; thus, the decrease in autotrophic iron- and sulfur-oxidizing bacteria could be one criterion for evaluating the successful revegetation of mine tailings^10^. To compare the different relative abundances of iron- and sulfur-oxidizing bacteria in the study field, 7 genera (*Acidithiobacillus*, *Alicyclobacillus*, *Ferrithrix*, *Leptospirillum*, *Sulfobacillus*, *Thiobacillus* and *Thiomonas*) were selected (Supplementary Fig. S6). Intriguingly, the total relative abundance of iron- and sulfur-oxidizing bacteria in the profile of BW was high, and revegetation indeed decreased the relative abundance of the iron- and/or sulfur-oxidizing bacteria (Supplementary Fig. S6). In particular, the genera of *Acidithiobacillus* and *Leptospirillum* were dominant in the profiles of BW with pH > 3, although these genera might be dominant in the metallic sulfides with pH < 3^24^,^26^,^47^,^48^. We suggest that the iron- and/or sulfur-oxidizing bacteria can spread over a wide pH range. In parallel, the relative abundances of *Thiobacillus* and *Ferrithrix* in profile horizons of 10–60 cm for CZ were higher than that of IC (t-test, *P* < 0.001, *P* = 0.003, respectively). All of these observations imply that *I. cylindrica* revegetation can more effectively inhibit the oxidation of polymetallic sulfides in the tailings. In parallel, the iron- and sulfur-oxidizing bacteria, which are usually found in oxidized tailings and acid mine drainage (AMD)/acid rock drainage (ARD), were also likely to participate in fixing CO_2_^50^,^51^ and might act as the main CO_2_-fixing bacteria in mine tailings. Although revegetation appeared to reduce the relative abundance of iron- and sulfur-oxidizing bacteria (Supplementary Fig. S6) while certain autotrophic bacteria did not increase significantly, the CO_2_-fixing bacteria did not decrease (Supplementary Fig. S5). We speculate that photosynthetic bacteria and/or other unknown chemoautotrophic bacteria became the main CO_2_-fixing bacteria instead of iron- and sulfur-oxidizing bacteria such as ammonia-oxidizing bacteria and denitrifiers.

In conclusion, our study highlighted the diversity and structure of bacterial communities in mine tailings with plant species. A shift in the diversity and structure of the microbial community occurred in the top 20 cm of the soil, which also showed a change in chemical properties such as pH, TN and TOC. More importantly, revegetation increased the nitrogen content through enriched communities of nitrogen-fixing bacteria; thus, IC might be more suitable for accumulating nutrients in mine tailings. We also found that IC possessed lower iron- and sulfur-oxidizing bacteria compared to CZ, which can more effectively inhibit the oxidation of polymetallic sulfides. Therefore, revegetation (especially IC revegetation) indeed promotes further development of the microbial community in tailings.

## Methods

### Site description and sampling

The copper mine tailings pond (30°55′N, 117°50′E, Supplementary Fig. S9) located in Tongling City, Anhui Province, East China covers a total surface area of 44 ha. The average annual rainfall in this area is 1346 mm, and the rainy season is from May to September. The average annual temperature is 16.2 °C, and the frost-free period lasts 237–258 days^52^.

*Imperata cylindrica* (IC) and *Chrysopogon zizanioides* (CZ) are distributed on the copper mine tailings, and bare wasteland (BW) was set as a control group. In this study field, two separate areas occupied by the two plant species were selected, and the soil samples beneath the vegetation were collected in May, 2013. The plant species selected were all of the same age and received no other management, and there was no interference between the different plants. Meanwhile, the tailings under vegetation in the study field and in bare wastelands were of the similar discharging time. For each plant vegetation type, three quadrats (area = 3 m × 3 m for each quadrat) were established as triplicate sampling sites. In each quadrat, three soil samples were randomly collected at each depth of soil profile (0–10 cm, 10–20 cm, 20–30 cm, 30–40 cm, 40–50 cm and 50–60 cm). These three subsamples at each depth from each quadrat were thoroughly homogenized to form a composite sample. Soil samples in sterilized plastic bags were immediately transported to the laboratory using an ice cooler and were stored at −80 °C before molecular analysis. One subsample was used for measurement of the soil chemical properties.

### Soil chemical property analysis

The pH was assessed with a water-to-tailing ratio of 5 by using a Mettler Toledo FE20 pH meter. The total organic carbon (TOC) was determined by the mass loss of ignition (LOI) in a muffle furnace at 550 ± 5 °C for 6 h. The total nitrogen (TN) content was measured using the Kjeldahl method. The water content was determined by a mass loss of approximately 10 g from a fresh tailings sample after drying at 105 °C for at least eight hours. Heavy metals (As, Cu, Fe, Pb and Zn) in the tailings were analyzed by inductively coupled plasma-atomic emission spectroscopy (ICP-AES, XSP Intrepid II, USA) after the samples had been digested by a mixture of nitric (68.0%), hydrofluoric (40.0%), and perchloric acid(72.0%).

### DNA extraction and pyrosequencing

Total genomic DNA in the samples was extracted by an modified method which combined SDS-based method^53^ and bead-beating protocols. Before extracting the total DNA, the tailings samples were pretreatment by high concentration EDTA mixture (100 mmol/L Tris-HCl (pH 8.0); 100 mmol/L phosphate (pH 8.0); 1 mol/L EDTA (pH 8.0) which was used to adjust pH and chelate heavy metal ion. The cell of samples was crushed by bead-beating protocols with 0.5g sterility beadings (approximately 0.5 mm) on a high-speed oscillator for 5 min in 600 μL of extraction buffer (100 mmol/L Tris-HCl (pH 8.0); 100 mmol/L phosphate (pH 8.0); 100 mmol/L EDTA (pH 8.0); 150 mmol/L NaCl; 1% (wt/vol) CTAB) before incubated at 37 °C for 90 min with 2 mg Lysozyme. It was further incubated at 65 °C for 30 min with 100 μL 20% SDS and 10 mg proteinase K before centrifugation at 8,000 × *g* for 20 min. The supernatant was mixed with 0.2 volumes 8 M potassium acetate (KAc) to remove polysaccharides followed by treatment with an equal volume of phenol-chloroform-isoamyl alcohol (25:24:1) to remove protein and cell debris. Residual phenol was removed by the addition of an equal volume of chloroform-isoamyl alcohol. Nucleic acids were precipitated from the supernatant by adding approximately an equal volume of isopropanol and maintaining the samples at 4 °C for at least 2 h. The solution was centrifuged at 14,000 × *g* for 20 min. Traces of isopropanol were completely removed by the addition of 70% (v/v) alcohol and centrifugation at 14,000 × *g* for 20 min. Extracted genomic DNA samples were dissolved in 50 μL of Tris-EDTA (TE) buffer and stored at −20 °C for further processing. The 16S rDNA genes of the samples were PCR-amplified for multiplexed pyrosequencing using bar-coded primers, and the V3–V5 regions of the bacterial 16S rDNA gene-specific primers were used (forward primer: CCG TCA ATT CMT TTG AGT TT and reverse primer: ACT CCT ACG GGA GGC AGC AG). Each reaction of the50 μl PCR mixture contained 50 ng DNA, 41 μl molecular biology-grade water, 5 μl 10× FastStart High Fidelity Reaction Buffer with 18 mM MgCl_2_, 1 μl dNTPs (10 mM each), 1 μl Fusion Primer A (10 μM), 1 μl Fusion Primer B (10 μM), and 1 μl FastStart High Fidelity Enzyme Blend (5 U/μl). PCR was initiated at 95 °C for 2 min; followed by 30 cycles of 95 °C for 20 s, 50 °C for 30 s, and 72 °C for 5 min; followed by a final extension at 72 °C for 10 min. The resulting amplicons were purified using Agencourt AMPure beads. Equal amounts of the PCR product from each sample were combined in a single tube to be run on a Roche GS FLX 454 pyrosequencing machine at Beijing Genomics Institute (BGI Shenzhen, China).

### RNA extraction and inverse transcription

To compare the different transcriptional *nifH* genes between plant species, the rhizosphere soil was gently shaken off the roots using Riley and Barber’s method^54^, and samples for RNA analysis were stored in RNAlater (1 g soil:1 ml RNAlater) (Ambion, Austin, TX). Total RNA in the RNAlater was thawed on ice and extracted following the mRNA extraction method of Mettel *et al.*^55^ RNA samples were inversely transcribed into cDNA with TransScript II All-in-one first-strand cDNA supermix for qPCR (TransGen Biotech, Beijing, PRC).

### Clone library construction of nifH gene and phylogenetic analysis

Clone libraries of *nifH* genes were constructed for DNA extracts from the top 0–20 cm of the tailings in the bare wasteland (BW) and of the tailings revegetated by *I. cylindrica* (IC) and *C. zizanioides* (CZ). Meanwhile, clone libraries of transcriptional *nifH* genes were constructed for cDNA from the rhizospheres (clone libraries of *nifH* transcripts and *nifH* genes were constructed from the soil samples at a depth of 0–20 cm of the tailings under IC and CZ vegetations) of IC and CZ. The *nifH* gene was amplified using primers F2/R6 referenced in a previous study^56^. Triplicate PCR products were pooled, purified with the EasyPure^®^ Quick Gel Extraction kit (TransGen Biotech, Beijing, PRC), and cloned using a pEASY-T3 Cloning kit (TransGen Biotech, Beijing, PRC). The correct insertion of *nifH* genes from recombinant clones was verified by PCR re-amplification with vector primers, and the resulting PCR products were subjected to enzymatic digestion using HhaI and MspI endonucleases (Thermo, US). Digested DNA fragments were resolved using electrophoresis on a 1.5% agarose gel, and the resulting restriction fragment-length polymorphism (RFLP) profiles were compared manually. Representative clones for each unique RFLP pattern were sequenced (BGI, Beijing, China).

### Quantification of functional genes by real-time PCR

To quantify *cbbL* and *nifH* gene numbers, primer pairs referenced in previous studies were used^56^,^57^,^58^. KAPA SYBR^@^ FAST qPCR Kits (KAPA Biosystems, Boston, US) were used for amplification, and the PCR program was set with reference to the specification. The amount of total bacterial 16S rDNA was determined by using primers 1055f (5′-ATG GCT GTC GTC AGC T-3′) and 1392r (5′-ACG GGC GGT GTG TAC-3′). The TaqMan probe 16S Taq1115 (6′FAM)-CAA CGA GCG CAA CCC-(TAMRA) was modified from the 1114f primer, amplification was carried out using the TaqMan Universal PCR Master Mix (ABI, America),and the PCR program was set with reference to the specification. All samples and standard reactions were performed in triplicate using a Stepone Real-Time PCR System (ABI, New York, America). Templates for the standard curve were prepared by 10-fold serial dilutions of linearized recombinant plasmids harboring an amplicon amplified from sample DNA. Recombinant plasmids containing one copy of the gene fragment were diluted from 1:10^2^ to 1:10^8^ and were used as the target for qPCR standard curves. Negative controls without template DNA were included in all experiments to exclude contamination. Each R^2^ value of each standard curve for each replicate was >0.990.

### Statistical analysis

Effective reads in the subsequent analyses were defined as sequences >200 bp in length, with an average quality score >25, without ambiguous base calls and with at least an 80% match to a previously determined 16S rRNA gene sequence using the Ribosomal Database Project (RDP) pyrosequencing pipeline (http:// pyro.cme.msu.edu/). OTU numbers were calculated based on 97% similarity of such effective reads, which were pre-clustered by scripts of the software Mothur (Version 1.31.2) within a 2-mismatch similarity. Scripts of software Mothur (Version 1.31.2) was used to obtain high-quality reads by filtering the chimaera sequences. First of all, all reads were assigned to corresponding samples by allowing one mismatch to the sample barcode and 2 mismatches to the adjacent PCR primer; Reads with an ambiguous base call or a homopolymer longer than 8 bp were removed. We then calculated the average quality score within a 50 bp window that was moved along the reads. When the average quality score dropped below 35, the reads was trimmed. The resulting reads shorter than 200 bp, or longer than 1000 bp were removed, and the redundant reads were eliminated by Mothur, and high-quality reads were generated. Secondly, all reads were aligned using a NAST-based sequence aligner to a custom reference based on the SILVA alignment. The reads that did not align to the anticipated region of the reference alignment were removed as chimaera. Reads that classified as “Cyanobacteria_Chloroplast”, “Mitochondria”, or “unknown” (those reads that could not be classified at the kingdom level) were removed. In addition, chimeric sequences identified by UCHIME algorithm were removed. Finally, all reads were classified using a Bayesian classifier with RDP database. In summary, a total of 196 985 high-quality reads were obtained out of 390 101 raw sequences. The operational taxonomic units (OTUs) were defined as sequences with >97% nucleotide sequence similarity based on DOTUR software, and the representative sequence within each OTU was extracted by using the software package mothur (Version 1.31.2) for subsequent analysis. The sequences have been deposited in GenBank short-read archive SRX648695.

We obtained 390 101 raw reads by pyrosequencing, and a total of 196 985 effective reads with sequences length >200 bp were retained after filtering. Operational taxonomic units (OTUs) were defined as sequences with at least 97% nucleotide sequence similarity based on DOTUR software. The *nifH* sequences were compared with those available in GenBank using the BLAST network service to determine their closest relatives. The aligned sequences were used to construct a phylogenetic tree using the neighbor-joining method in MEGA 4. Bootstrap confidence values were obtained based on 1000 replicates. The nucleotide *nifH* sequences have been deposited in the GenBank Data Library under accession numbers KR865982-KR866018. Chimeric sequences identified by Chimera (version 1.6) were removed.

ANOVA was used to compare the difference among IC, CZ and BW in any index used in the study. Redundancy analysis (RDA) was used to identify the abiotic factors that are most important to the bacterial community composition in R v.3.1.0 using the ‘rda’ and ‘envfit’ function of the vegan package, allowing for full permutation of the raw data and Monte Carlo tests with 999 permutations using the ‘permutest’ function of the vegan package. Variation partitioning analysis (VPA) was used to determine the effects of soil characteristics, plant species, and depth and interactions between these parameters on the structure of the bacterial community using the ‘varpart’ function of the vegan package. Bray-Curtis distances calculated for the total community analyses were clustered with Ward’s minimum variance clustering method in the R environment using the ‘hclust’ function of the cluster package; the results were visualized using non-metric multidimensional scaling (NMDS) plots.

## Additional Information

**How to cite this article**: Li, Y. *et al.* Ecological restoration alters microbial communities in mine tailings profiles. *Sci. Rep.* **6**, 25193; doi: 10.1038/srep25193 (2016).

## Supplementary Material

Supplementary Information
