# Supplementary material for: Ecological restoration alters microbial communities in mine tailings profiles
Source: Sci Rep. 2016 Apr 29;6:25193. doi: 10.1038/srep25193 (PMC4850430; doi:10.1038/srep25193)
Supplement: Supplementary Information [file srep25193-s1.pdf]

Supplementary information for

Title: Ecological restoration alters microbial communities in mine tailings profiles

Author names: Yang Li[1], Zhongjun Jia[2], Qingye Sun[1,*], Jing Zhan[1], Yang Yang[1], Dan Wang[1]

[1]School of Resources and Environmental Engineering, Anhui University, Hefei, Anhui Province, China

[2]State Key Laboratory of Soil and Sustainable Agriculture, Institute of Soil Science, Chinese Academy of Sciences, Nanjing, Jiangsu Province, China

*Correspondence should be addressed to Qingye Sun at

E-mail: *sunqingye@ahu.edu.cn*; Tel/Fax: +86-551-6386-1882

This file includes:

1. Supplementary Table S1-S3
2. Supplementary Figure S1-S9

Supplementary Tables

Supplementary Table S1 F values of pairwise comparisons of Bray-Curtis similarity index among different plant covered of profiles by one-way non-parametric multivariate analysis of variance (NPMANOVA) analysis. Single asterisks values indicated significant difference at the level < 0.05 and double asterisks values indicated significant difference at the level < 0.01; n.s. indicated non-significant differences; non-significant differences was found among different plant species in horizons 20-60cm (data not shown).

horizon		BW	VZ	IC
0-10cm	BW		16.787^{**}	14.179^{**}
	VZ			n.s.
	IC			
10-20cm	BW		8.233^{**}	11.490^{**}
	VZ			n.s.
	IC			

Supplementary Table S2 The Pearson correlation(r) between main identified bacterial taxonomic groups, i.e. phyla *Acidobacteria*, *Actinobacteria*, *Bacteroidetes* and *Firmicutes*, classes *Alphaproteobacteria*, *Betaproteobacteria*, *Gammaproteobacteria* and *Deltaproteobacteria*, (within *Proteobacteria* phylum) and environment parameters. Single asterisk show the significant difference at the level < 0.05, and double asterisk show the significant difference at the level < 0.01.

	LOI	TN	pH	Water con.	As	Cu	Fe	Pb	Zn
Acidobacteria	0.384**	0.286*	0.247	-0.03	-0.507**	0.164	0.181	0.120	0.424**
Actinobacteria	0.361**	0.449**	-0.189	0.225	-0.166	0.126	0.548**	0.431**	-0.068
Bacteroidetes	-0.212	-0.391**	0.434**	-0.256	-0.069	0.156	-0.397**	0.188	0.109
Firmicutes	-0.025	0.207	-0.647**	0.396**	0.260	-0.231	0.496**	-0.038	-0.561**
Nitrospira	-0.166	0.103	-0.567**	0.122	0.442**	-0.114	0.125	-0.294*	-0.298*
Alphaproteobacteria	0.632**	0.531**	0.049	0.049	-0.496**	0.168	0.359**	0.101	0.389**
Betaproteobacteria	-0.252	-0.295*	0.164	-0.195	0.156	-0.078	-0.537**	-0.308*	0.106
Gammaproteobacteria	-0.263	-0.131	-0.294*	0.065	0.461**	-0.278*	-0.106	-0.374**	-0.284*
Deltaproteobacteria	0.280*	0.145	0.322*	-0.251	-0.206	0.145	-0.033	-0.087	0.419**

Supplementary Table S3 Diversity and predicted richness of *nifH* sequences from the clone libraries*

	clones number	OTUs number	Coverage (%)	Shannon index (*H'*)	Simpson's index	Pielou evenness index
BW(DNA)	108	12	100	2.13	0.85	0.45
IC(DNA)	108	27	97.2	3.03	0.94	0.65
VZ(DNA)	107	27	94.4	3.05	0.94	0.65
IC(cDNA)	106	16	98.1	2.29	0.86	0.49
VZ(cDNA)	108	17	100	2.57	0.90	0.55

*OTUs were defined by a 3%difference in the nucleic acid sequence alignment for the *nifH* gene.

Supplementary figures:

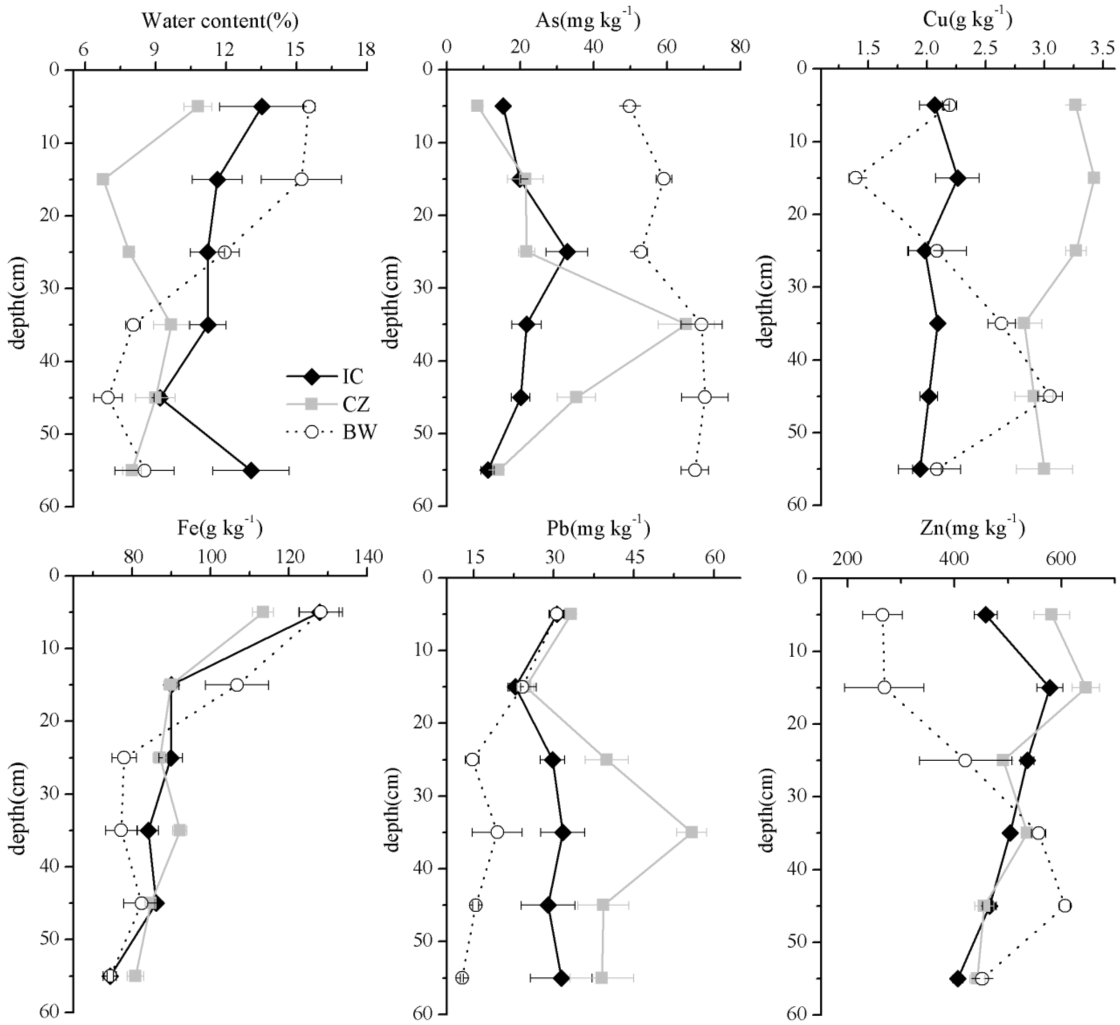

Supplementary Fig.S1 Physico-chemical properties of 6 horizons from different plant types of profiles. The error bars showed the standard error of relative abundance of the three subsamples for each tailings sample. IC and CZ: tailings revegetated by *Imperata cylindrica* and *Chrysopogon zizanioides*, BW: bare wasteland.

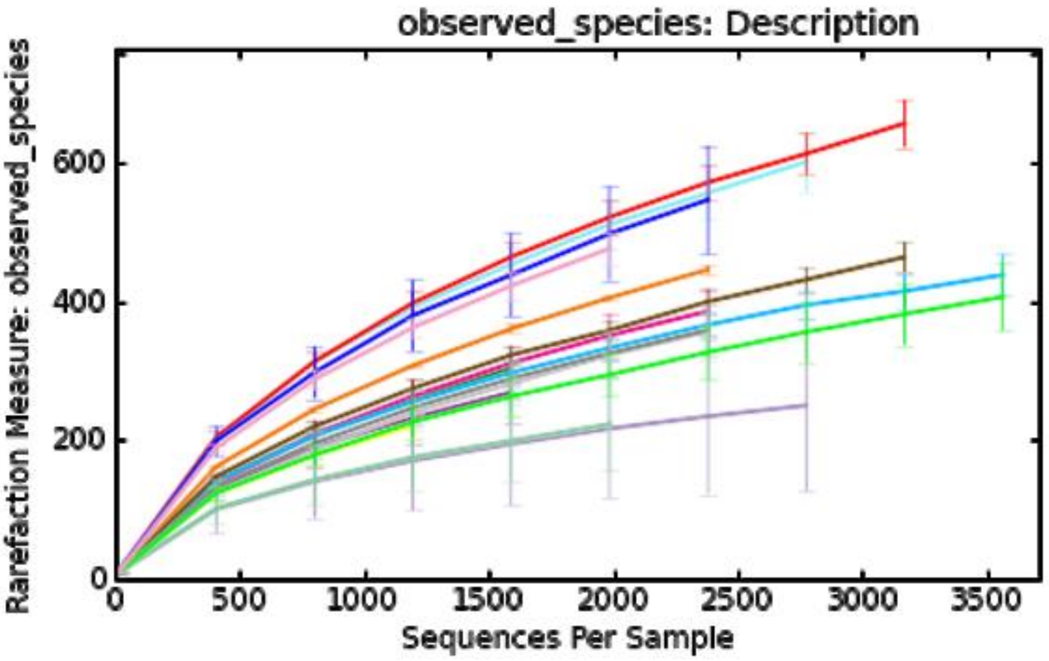

Supplementary Fig. S2 Rarefaction curve of OTUs（observed_species）recovered from mine tailings.

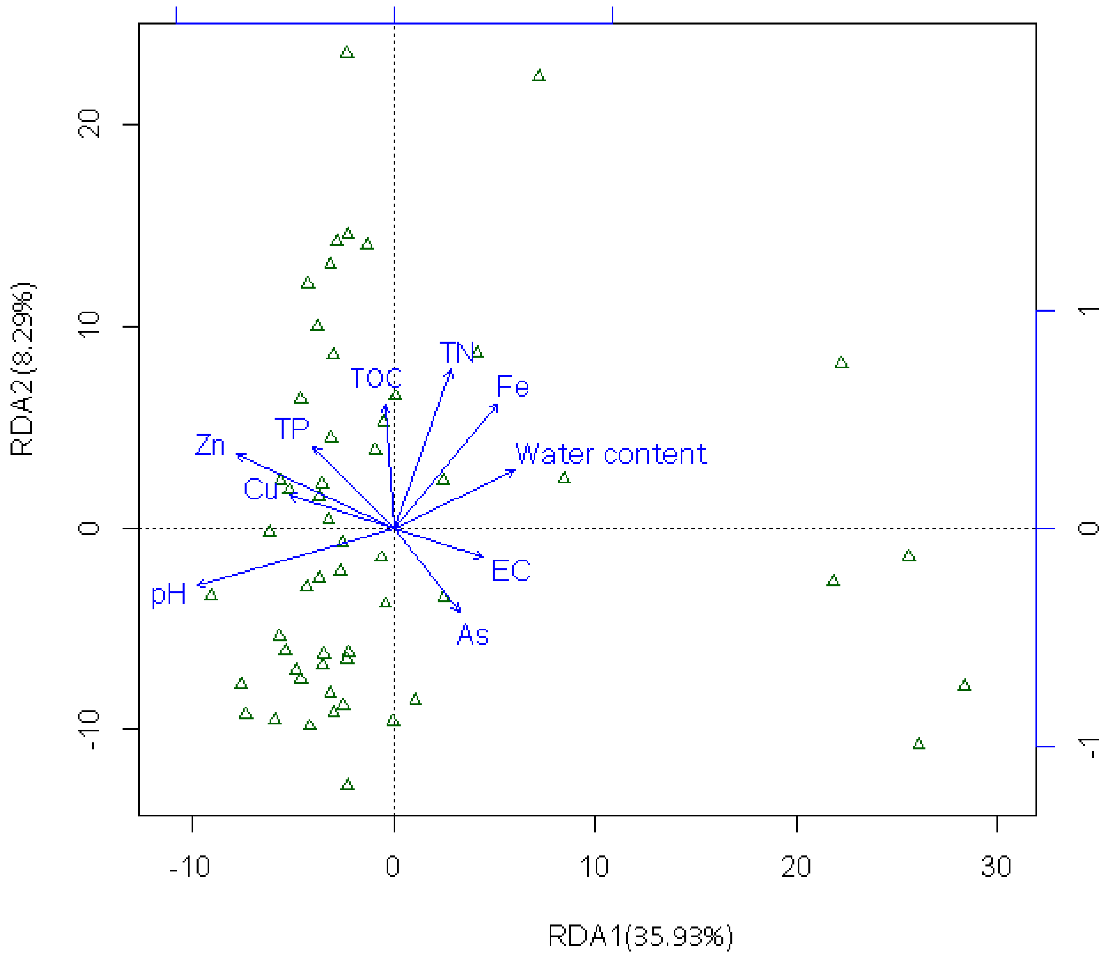

Supplementary Fig.S3 Redundancy analysis (RDA) for 6 horizons of different plant species with forward selection of predictor variables followed by Monte Carlo permutations (999 permutations). Solid arrows represent predictor (chemical) variables significantly associated (P<0.05) with the variation in the bacterial community structure.

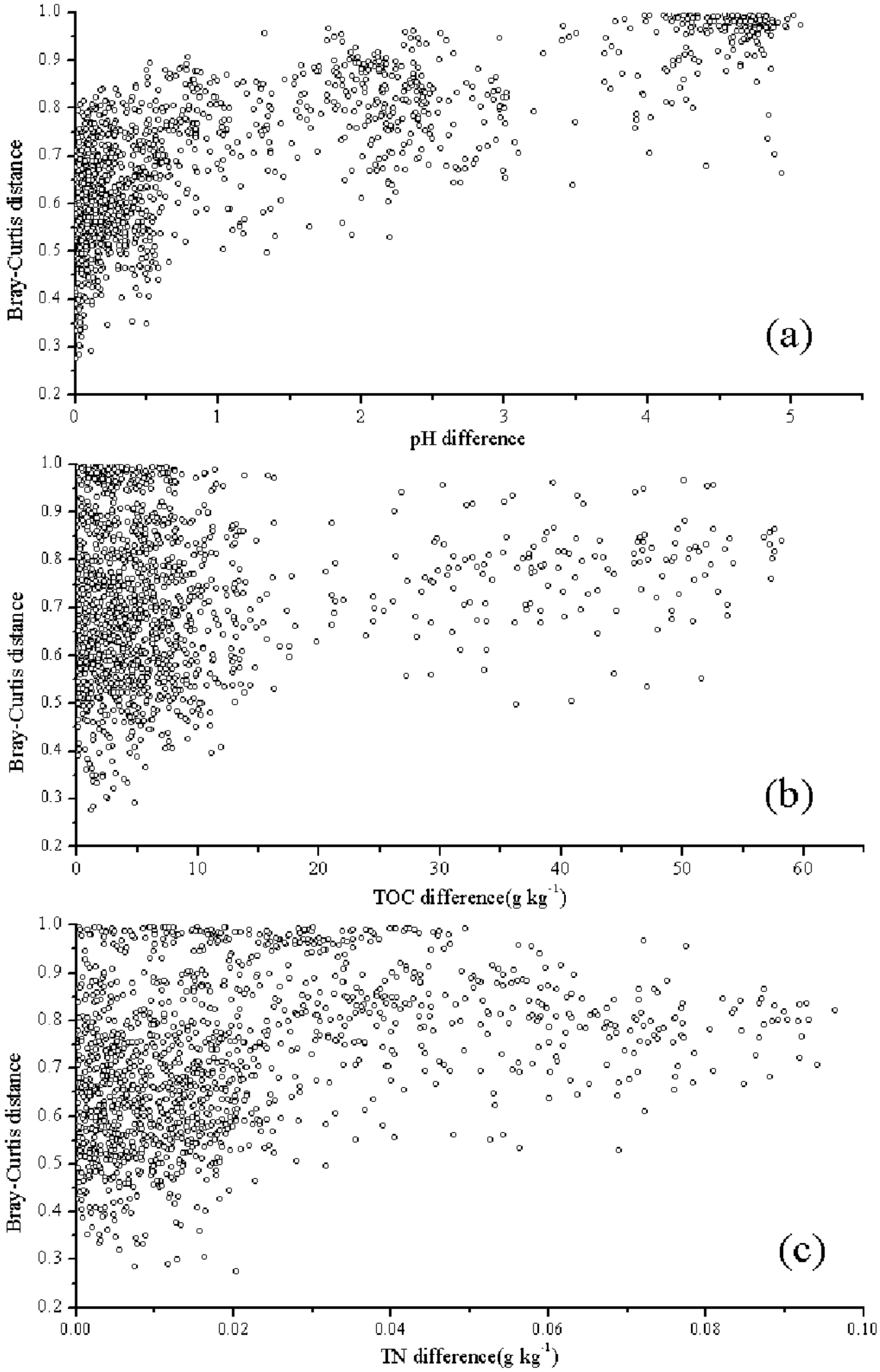

Supplementary Fig. S4 Relationships between (a) pH, (b) TOC and (c) TN and bacterial community diversity (Bray-Curtis similarity on the basis of phylotype composition).

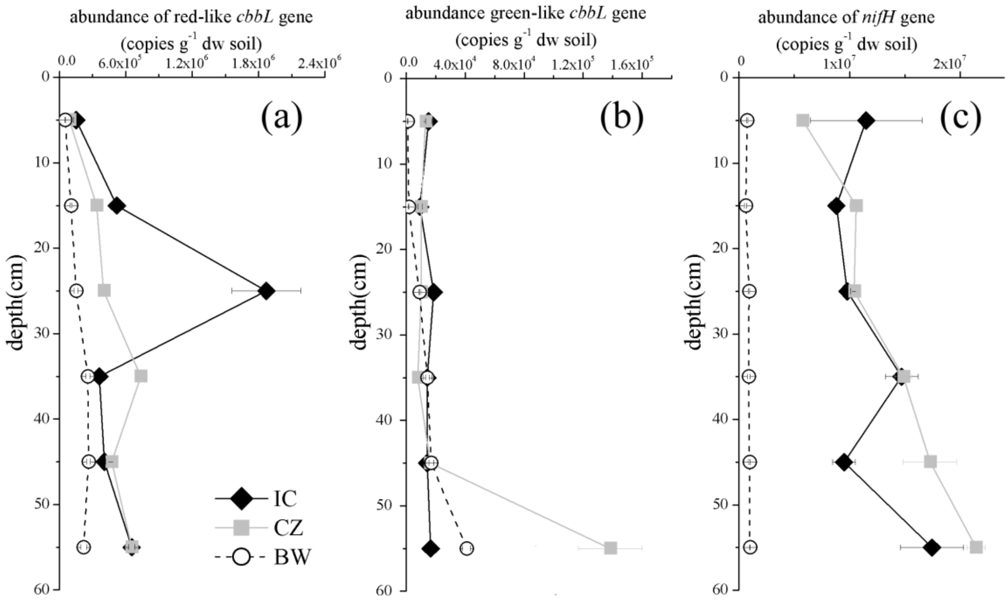

Supplementary Fig.S5 Abundance of *cbbL* (red-like(a) and green-like(b)) and *nifH* (c) genes along the profiles (copies g^{-1}dry soil, n=3). Error bars indicated standard error. IC and CZ: tailings revegetated by *I. cylindrica* and *C. zizanioides*, BW: bare wasteland. The *cbbL* gene encoded the large subunit of form I ribulose 1,5-bisphosphate carboxylase/oxygenase (RubisCO) which was the first and rate-limiting step in Calvin-Benson-basham (CBB) reductive pentose phosphate pathway, in which form I RubisCO can be divided into two major groups, green-like and red-like, in terms of the amino acid compositions and length of branches in the phylogenetic tree. And *nifH* gene encoded dinitrogenase reductase which is a key enzyme of nitrogen fixation.

(a)

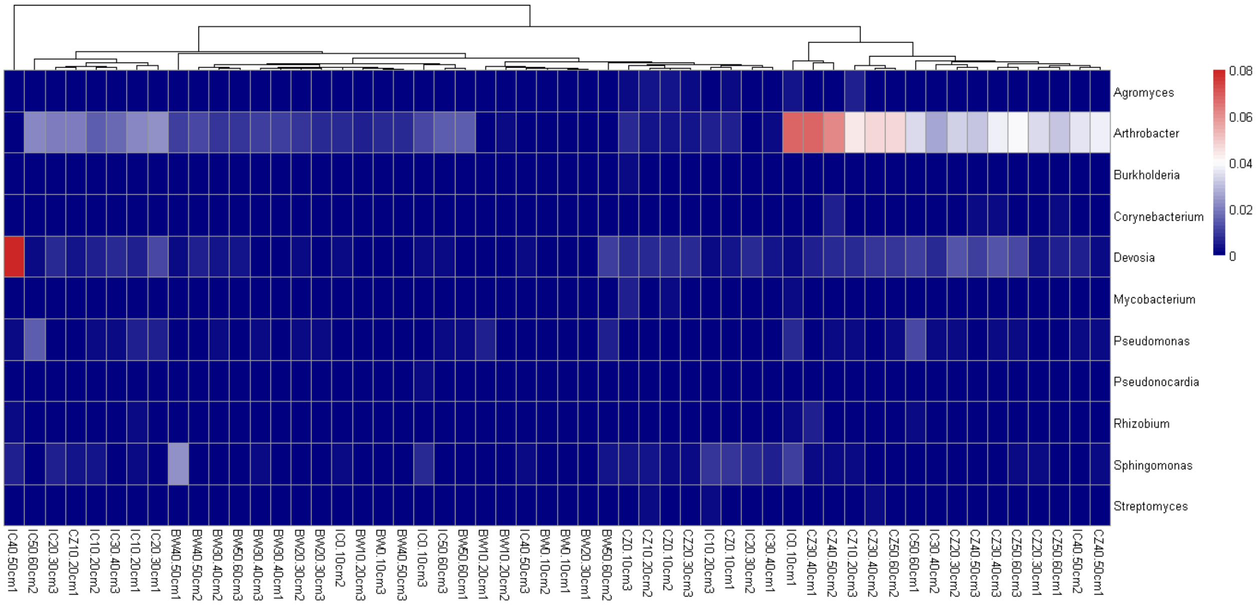

(b)

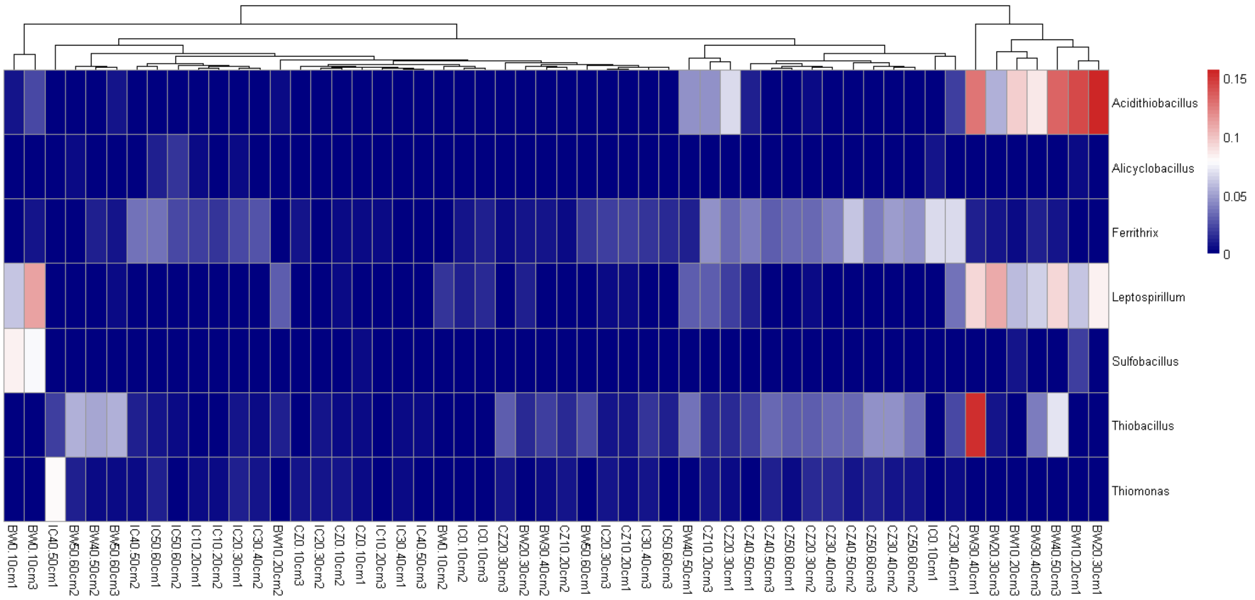

Supplementary Fig.S6 Heat map for nitrogen- fixing genera(a), and iron- & sulfur-oxidizing bacteria (b) in 6 horizons of different plant species. The genera of *Agromyces*[1, 2], *Arthrobacter*[1-4], *Burkholderia*[5], *Corynebacterium*[6], *Devosia*[7], *Mycobacterium*[4, 8], *Pseudomonas*[9], *Pseudonocardia*[2], *Rhizobium*[9], *Sphingomonas*[10] and *Streptomyces*[4] were likely to be involved in nitrogen fixation. 7 genera (*Acidithiobacillus*[11], *Alicyclobacillus*[12], *Ferrithrix*[13], *Leptospirillum*[11], *Sulfobacillus*[12, 14], *Thiobacillus*[11] and *Thiomonas*[15]) related with iron- and sulfur- oxidizing were found in this study.

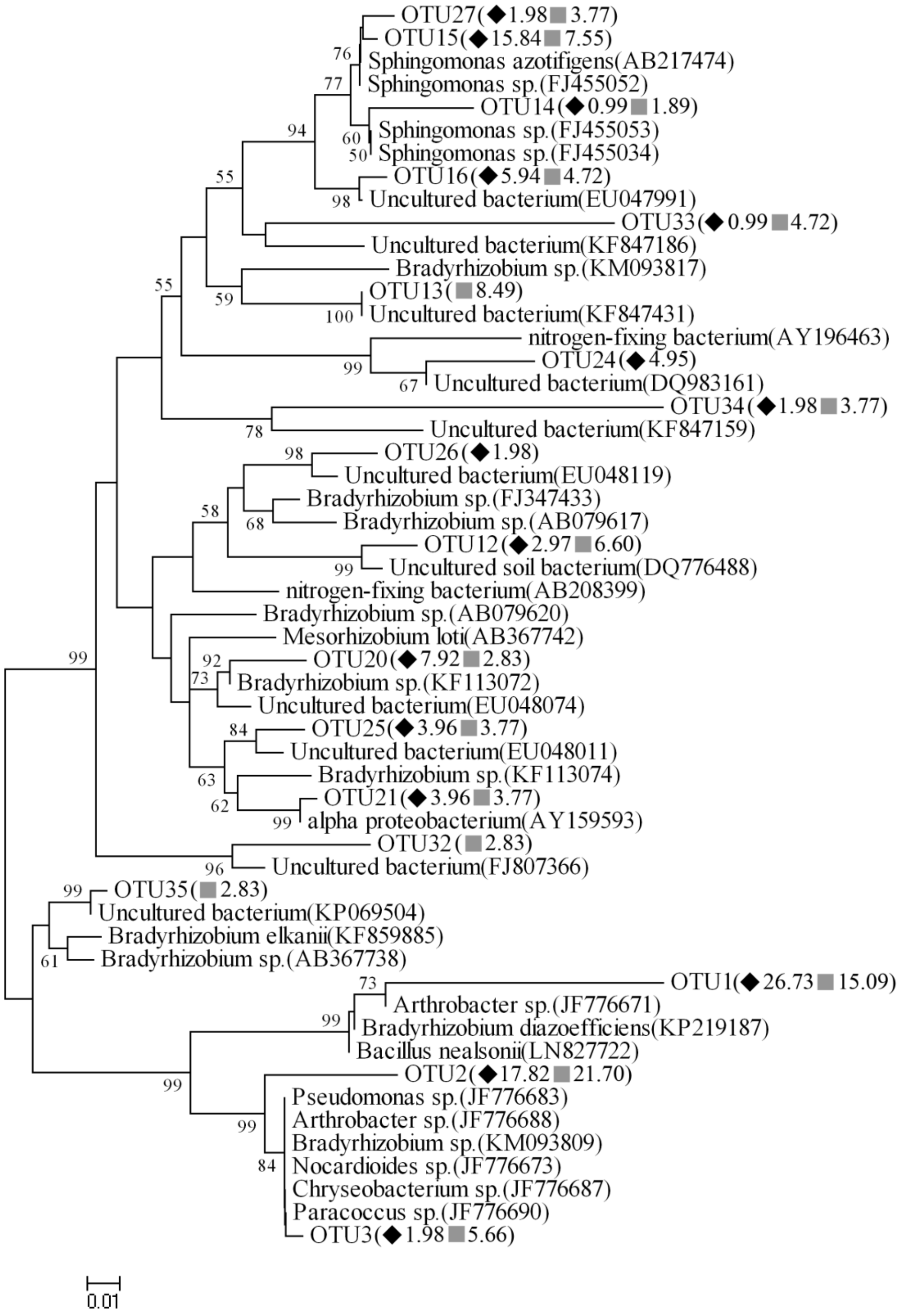

Supplementary Fig.S7 Phylogenetic tree of transcriptional *nifH* sequences in rhizosphere. Clones from the present study are marked with rhizosphere of *I. cylindrica* (IC) and rhizosphere of *C. zizanioides* (CZ). Additional symbols show the relative frequency (%) of a sequence in their respective clone libraries (◆, IC; ■, CZ). Bootstrap values of >50% are exhibited at branch points.

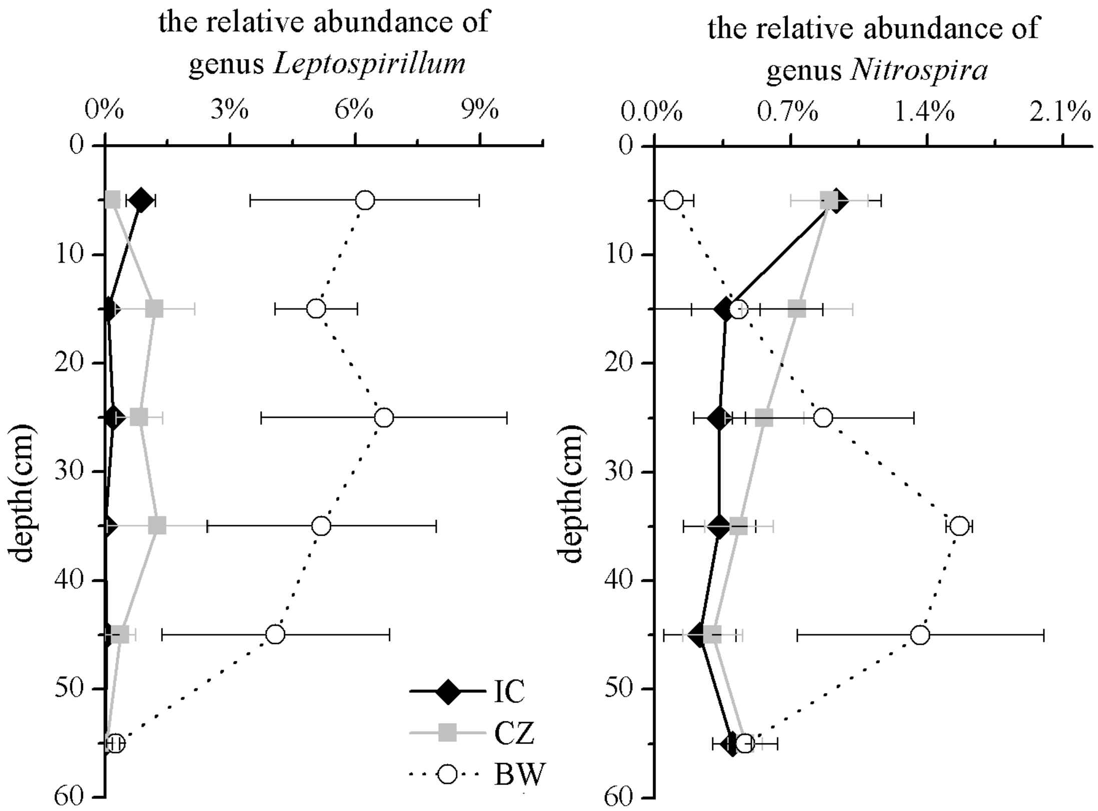

Supplementary Fig.S8 Relative abundance (percentage) of genera of *Leptospirillum* and *Nitrospira* both of which belonging to phylum *Nitrospira.* The error bars showed the standard error of relative abundance of the three subsamples for each tailings sample. IC and CZ: tailings revegetated by *I. cylindrica* and *C. zizanioides*, BW: bare wasteland.

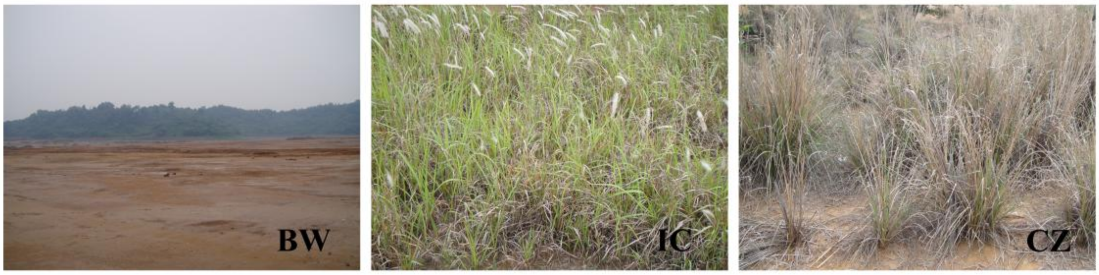

Supplementary Fig. S9 Examples of the colonizer plant species in the Shuimuchong tailings pond. BW: bare wasteland; IC and CZ: tailings revegetated by *I. cylindrica* and *C. zizanioides*.

References

1. Sellstedt, A. & Richau, K.H. Aspects of nitrogen-fixing *Actinobacteria*, in particular free-living and symbiotic Frankia. *FEMS Microbiol. Lett.* **342**, 179-186 (2013).
2. Gtari, M., Ghodhbane-Gtari, F., Nouioui, I., Beauchemin, N. & Tisa, L.S. Phylogenetic perspectives of nitrogen-fixing actinobacteria. *Arch. Microbiol.* **194**, 3-11 (2012).
3. Smyk, B. Fixation of atmospheric nitrogen by the strains of *Arthrobacter*. *Zentralblatt für Bakteriologie, Parasitenkunde, Infektionskrankheiten und Hygiene. Zweite naturwissenschaftliche Abt.: Allgemeine, landwirtschaftliche und technische Mikrobiologie* **124**, 231 (1970).
4. Young, J. Phylogenetic classification of nitrogen-fixing organisms. *Biological nitrogen fixation*, 43-86 (1992).
5. Chen, W.-M. et al. Legume symbiotic nitrogen fixation by β-proteobacteria is widespread in nature. *J. Bacteriol.* **185**, 7266-7272 (2003).
6. Berndt, H., Lowe, D.J. & Yates, M.G. The Nitrogen-Fixing System of *Corynebacterium autotrophicum*. *Eur. J. Biochem.* **86**, 133-142 (1978).
7. Rivas, R. et al. Description of *Devosia neptuniae* sp. nov. that nodulates and fixes nitrogen in symbiosis with Neptunia natans, an aquatic legume from India. *Syst. Appl. Microbiol.* **26**, 47-53 (2003).
8. Biggins, D. & Postgate, J. Nitrogen fixation by cultures and cell-free extracts of *Mycobacterium flavum* 301. *Microbiology* **56**, 181-193 (1969).
9. Deryło, M. & Skorupska, A. Enhancement of symbiotic nitrogen fixation by vitamin-secreting fluorescent *Pseudomonas*. *Plant Soil* **154**, 211-217 (1993).
10. Videira, S.S., De Araujo, J.L.S., Rodrigues, L.D.S., Baldani, V.L.D. & Baldani, J.I. Occurrence and diversity of nitrogen-fixing *Sphingomonas* bacteria associated with rice plants grown in Brazil. *FEMS Microbiol. Lett.* **293**, 11-19 (2009).
11. Dold, B. Evolution of Acid Mine Drainage formation in sulphidic mine tailings. *Minerals* **4**, 621-641 (2014).
12. Karavaiko, G.I. et al. Reclassification of '*Sulfobacillus thermosulfidooxidans* subsp. thermotolerans' strain K1 as *Alicyclobacillus tolerans* sp. nov. and *Sulfobacillus disulfidooxidans* Dufresne *et al*. 1996 as *Alicyclobacillus disulfidooxidans* comb. nov., and emended description of the genus *Alicyclobacillus*. *Int. J. Syst. Evol. Micr.* **55**, 941-947 (2005).
13. Johnson, D.B., Bacelar-Nicolau, P., Okibe, N., Thomas, A. & Hallberg, K.B. *Ferrimicrobium acidiphilum* gen. nov., sp. nov. and *Ferrithrix thermotolerans* gen. nov., sp. nov.: heterotrophic, iron-oxidizing, extremely acidophilic actinobacteria. *Int. J. Syst. Evol. Micr.* **59**, 1082-1089 (2009).
14. Stott, M., Watling, H., Franzmann, P. & Sutton, D. The role of iron-hydroxy precipitates in the passivation of chalcopyrite during bioleaching. *Miner. Eng.* **13**, 1117-1127 (2000).
15. Coupland, K. et al. Oxidation of iron, sulfur and arsenic in mine waters and mine wastes: an important role for novel *Thiomonas* spp. (2004).
